# Ultrasound renal denervation: the future of hypertension management?

**DOI:** 10.1186/s43044-023-00387-w

**Published:** 2023-07-03

**Authors:** Laiba Ali, Hareer Fatima, Faiza Riaz, Muhammad Saqlain Mustafa, Burhanuddin Sohail Rangwala, Hussain Sohail Rangwala

**Affiliations:** Department of Medicine, Jinnah Sindh Medical University, Iqbal Shaheed Rd, Karachi, Pakistan

**Keywords:** Hypertension, Renal denervation, Radiofrequency energy

## Abstract

**Background:**

Hypertension, a leading cause of global mortality and morbidity, affects approximately 1.28 billion adults worldwide, with most cases occurring in low- and middle-income countries. Despite several methods for managing mild to moderate hypertension, effective management of severe or resistant hypertension remains challenging. Renal denervation, a promising non-pharmacological technique, has emerged as a potential solution.

**Main body:**

Renal denervation works by modifying the renal sympathetic nerve supply through techniques such as ultrasound, radiofrequency energy, or injection of neurolytic agents, reducing blood pressure. Clinical trials, including the RADIANCE series, have shown consistent effectiveness of ultrasound renal denervation in lowering blood pressure, especially in patients who were previously unresponsive to anti-hypertensive medications. After a follow-up of 2 months, mean ambulatory systolic blood pressure during the daytime decreased significantly in the ultrasound renal denervation group compared to the sham group. However, further research is needed to determine renal denervation's long-term safety and efficacy.

**Conclusions:**

In conclusion, renal denervation holds great potential in improving the treatment of uncontrolled or resistant hypertension treatment, but more investigations and trials are necessary to establish its effectiveness and safety.

## Background

Hypertension is a leading cause of both mortality and morbidity globally. It is estimated that 1.28 billion adults between the ages of 30–79 years suffer from hypertension worldwide, with the majority of cases (two-thirds) occurring in low- and middle-income countries. Approximately half (46%) of adults with hypertension are unaware of their condition. While several methods are available for managing mild to moderate hypertension, effective management of severe or resistant hypertension remains a challenge. A promising new non-pharmacological technique called Renal Denervation has emerged to address this issue. The Renal Denervation technique modifies the renal sympathetic nerve supply and introduces heat energy to the sympathetic nerves surrounding the kidneys. This results in a reduction in blood pressure. This technique can be used with anti-hypertensive drugs or as a standalone treatment. [1, 2].

## Main body

The renal denervation technique consists of three different methods by which it can be carried out. These include using ultrasound, radiofrequency energy, and injecting neurolytic agents into the vascular tissues surrounding the kidneys. This technique is still relatively new, and more investigations and trials must be done [3].

The effectiveness of Ultrasound Renal Denervation has been investigated in the guise of 3 separate multicenter, international, blinded, randomized, sham-controlled trials, that are as follows: 1. Endovascular ultrasound renal denervation to treat hypertension (RADIANCE-HTN SOLO) [4]; 2. Ultrasound renal denervation for hypertension resistant to a triple medication pill (RADIANCE-HTN TRIO) [5], and 3—endovascular Ultrasound Renal Denervation to Treat Hypertension: The (RADIANCE II) Randomized Clinical Trial [6]. In the first trial, the target population had patients who were previously consuming anti-hypertensive drugs but were not consuming them. In the second trial, the target population was patients who were resistant to more than three anti-hypertensive drugs even after shifting to a single pill made of a combination of three anti-hypertensive drugs. The third trial in the RADIANCE series was done on patients who had high blood pressure despite taking up to two anti-hypertensive drugs; this trial aimed to assess the effectiveness of Ultrasound Renal Denervation without the influence of anti-hypertensive drugs. These trials, especially the RADIANCE-HTN TRIO trial, explicitly tell us how patients with no change with various anti-hypertension medications can decrease their blood pressure after undergoing Ultrasound Renal Denervation. Thus, in itself, it proves this technique's importance in treating resistant or uncontrolled hypertension. All three studies of the before-mentioned clinical trials involved patients aged 18 to 75 years. In total, 506 patients were investigated in the three studies.

Ajay and his colleagues have pooled the data from these three clinical trials [7]. In light of the pooled data, it can be said that Ultrasound Renal Denervation consistently lowered blood pressure regardless of the severity of HTN in sham-controlled trials created with a goal of a 2-month end period to measure medication across groups that were randomly generated uniformly. After a follow-up of 2 months, mean ambulatory systolic Blood pressure during daytime decreased by 8.5 mm Hg from the pre-randomization baseline to 141.8 (13.8) mm Hg after an Ultrasound Renal Denervation vs. a drop in blood pressure to 147.9 (14.6) mm Hg after sham which is a decrease of only 2.9 mm Hg [7]. Hence this significantly proves the effectiveness of Ultrasound Renal Denervation techniques and their future potential in treating uncontrolled or resistant hypertension.

## Conclusions

In conclusion, hypertension is a significant global health concern, with many cases going undiagnosed. Renal denervation, a non-pharmacological technique that modifies the renal sympathetic nerve supply, has emerged as a promising solution for managing severe or resistant hypertension and has shown consistent effectiveness in decreasing blood pressure in randomized clinical trials. Although further research is required to determine its long-term safety and efficacy, ultrasound renal denervation holds great potential in improving the treatment of hypertension.

## Data Availability

Not applicable.

## Abbreviations

RADIANCE-HTN SOLO: Endovascular ultrasound renal denervation to treat hypertension
RADIANCE- HTN TRIO: Ultrasound renal denervation for hypertension resistant to a triple medication pill
RADIANCE II: Endovascular Ultrasound Renal Denervation to Treat hypertension
